# Identification of Disease-Promoting *HLA* Class I and Protective Class II Modifiers in Japanese Patients with Familial Mediterranean Fever

**DOI:** 10.1371/journal.pone.0125938

**Published:** 2015-05-14

**Authors:** Michio Yasunami, Hitomi Nakamura, Kazunaga Agematsu, Akinori Nakamura, Masahide Yazaki, Dai Kishida, Akihiro Yachie, Tomoko Toma, Junya Masumoto, Hiroaki Ida, Tomohiro Koga, Atsushi Kawakami, Katsumi Eguchi, Hiroshi Furukawa, Tadashi Nakamura, Minoru Nakamura, Kiyoshi Migita

**Affiliations:** 1 Department of Clinical Medicine, Institute of Tropical Medicine, Nagasaki University, Nagasaki, Japan; 2 Department of Infection and Host Defense, Graduate School of Medicine, Shinshu University, Matsumoto, Japan; 3 Department of Medicine (Neurology and Rheumatology), Shinshu University School of Medicine, Matsumoto, Japan; 4 Department of Pediatrics, Kanazawa University, Kanazawa, Japan; 5 Department of Pathogenomics, Ehime University Graduate School of Medicine, To-on, Japan; 6 Department of Rheumatology, Kurume University School of Medicine, Kurume, Japan; 7 First Department of Internal Medicine, Nagasaki University School of Medicine, Nagasaki, Japan; 8 Department of Rheumatology, Sasebo City General Hospital, Sasebo, Japan; 9 Department of Rheumatology, National Hospital Organization Sagamihara Hospital, Sagamihara, Japan; 10 Department of Rheumatology, Kumamoto Shinto General Hospital, Kumamoto, Japan; 11 Clinical Research Center, National Hospital Organization Nagasaki Medical Center, Omura, Japan; University of Thessaly, Faculty of Medicine, GREECE

## Abstract

**Objectives:**

The genotype-phenotype correlation of *MEFV* remains unclear for the familial Mediterranean fever (FMF) patients, especially without canonical *MEFV* mutations in exon 10. The risk of FMF appeared to be under the influence of other factors in this case. The contribution of *HLA* polymorphisms to the risk of FMF was examined as strong candidates of modifier genes.

**Methods:**

Genotypes of *HLA-B* and *-DRB1* loci were determined for 258 mutually unrelated Japanese FMF patients, who satisfied modified Tel-Hashomer criteria, and 299 healthy controls. The effects of carrier status were evaluated for the risk of FMF by odds ratio (OR). The *HLA* effects were also assessed for clinical forms of FMF, subsets of FMF with certain *MEFV* genotypes and responsiveness to colchicine treatment.

**Results:**

The carriers of B*39:*01* were increased in the patients (OR = 3.25, p = 0.0012), whereas those of DRB1*15:*02* were decreased (OR = 0.45, p = 0.00050), satisfying Bonferroni’s correction for multiple statistical tests (n = 28, p<0.00179). The protective effect of DRB1*15:*02* was completely disappeared in the co-existence of B*40:*01*. The *HLA* effects were generally augmented in the patients without a canonical *MEFV* variant allele M694I, in accordance with the notion that the lower penetrance of the mutations is owing to the larger contribution of modifier genes in the pathogenesis, with a few exceptions. Further, 42.9% of 14 colchicine-resistant patients and 13.5% of 156 colchicine-responders possessed B*35:*01* allele, giving OR of 4.82 (p = 0.0041).

**Conclusions:**

The differential effects of *HLA* class I and class II polymorphisms were identified for Japanese FMF even in those with high-penetrance *MEFV* mutations.

## Introduction

Familial Mediterranean fever (FMF) has been considered to be an autosomal recessive trait which is characterized by self-limiting recurrent fever and serositis (OMIM #249100),[1] and categorized to an autoinflammatory disease.[2] *MEFV*, which was identified as the responsible gene for FMF, encodes cytosolic protein pyrin (also known as marenostrin) which regulates the activity of NLRP3 inflammasome.[3] Two hundred and ninety six sequence variants in *MEFV* gene have been registered to “Infevers” database (http://fmf.igh.cnrs.fr/ISSAID/infevers/) as of September 1st, 2014, including hot spots for pathogenic amino acid substitutions in the C-terminus region of the protein. Recently, dominant form (OMIM #134610) of the disease was reported,[4] and overlapping and continuum to other autoinflammatory diseases were proposed.[5] We performed a nation-wide surveillance of FMF in Japan and found that only M694I is commonly identified among hot spot mutations in the exon 10, homozygotes of which comprise as many as 10% of Japanese FMF.[6] We also noticed that more than one third of the patients did not carry two copies of pathological mutations in their *MEFV* gene.[6] It is possible that the indistinct genotype-phenotype correlation observed in Japanese FMF is owing to the effect of modifier genes. Genes in the *HLA* region are candidates for the modifier genes because they are involved in various inflammatory diseases such as ankylosing spondylitis,[7] rheumatoid arthritis,[8] and Behçet’s disease.[9]

In the present study, we examined the effect of carrier status of *HLA-B* and-*DRB1* alleles on FMF by the comparison of the frequencies between patients and non-disease control individuals by employing the probands of FMF pedigree and sporadic FMF cases who were captured by our previous national surveillance study.[6] HLA effects on clinical forms of FMF, subsets of FMF with certain *MEFV* genotypes and poor responders to colchicine treatment were also evaluated.

## Methods

### Study population

Blood samples were donated by the probands of FMF pedigree or sporadic cases of FMF, who satisfied the modified Tel-Hashomer criteria for FMF with either “typical” or “incomplete” attacks,[10] after the acquisition of written informed consent from each patient. Information about clinical feature was reported by health care providers participated to national surveillance study in Japan as reported previously;[6] in brief, epidemiological data (including gender, consanguinity of parents, familial history and age of onset of inflammation signs) and major clinical data (including fever, thoracic, abdominal, articular, cutaneous signs, duration and frequency of episodes, presence of amyloidosis, and response to colchicine) were provided by a standard form. Differential diagnosis between typical and incomplete attacks was made according to the following criteria: a typical FMF attack is defined as 3 or more episodes of generalized peritonitis, or monoarthritis of the hip, knee or ankle lasting 12 hours to 3 days with a fever of 38°C or more, while an incomplete attack is defined as a fever less than 38°C of ill-defined duration (from 6 hours to 1 week), no sign of peritonitis or localized abdominal sign during abdominal attack and atypical distribution of arthritis.[6] Genotype of *MEFV* was determined during a diagnostic procedure;[6] E84K, L110P, E148Q, R202Q, E225K, G304R, R354Q, P369S, R408Q, R410H, S503C, M694I and P751L were identified. All the study procedures were approved by institutional review boards of National Hospital Organization Nagasaki Medical Center and Nagasaki University.

### *HLA-B* and -*DRB1* genotyping

DNA was extracted from the blood sample and subjected to *HLA-B* and-*DRB1* genotype determination by WAKFlow HLA typing kit (Wakunaga Pharmaceutical, Osaka, Japan) based on the reverse sequence-specific oligonucleotide probes method coupled with xMAP technology designed for use with the Luminex system (Luminex Japan, Tokyo, Japan).

### Statistics

The risk/protective effects of carrier status of *HLA* alleles were evaluated by odds ratio (OR) obtained by the comparison of frequencies between patients and controls. *HLA* genotype data of 299 healthy volunteers, who were staff of National Hospitals, were used as controls in the present study. Independency or interaction between *HLA* factors was assessed by subgroup analysis after the stratification by carrier status of *HLA* alleles of interest. Further, the *HLA* effect on the responsiveness to colchicine treatment was evaluated by the comparison of *HLA* carrier status between poor responders and responders. All the statistical analyses were performed using STATA12 (StataCorp, College Station, TX, USA).

## Results

### Clinical feature of patients with FMF

Two hundred fifty eight mutually unrelated patients with FMF were enrolled in the present study. Among them, 149 met the criteria for “typical” FMF (FMF with typical attacks) and the other 109 were classified as “incomplete” FMF patients (patients with incomplete FMF attacks). *MEFV* M694I was carried by 85 FMF patients (32.9%) and 159 patients (61.6%) possessed two copies of *MEFV* sequences with detectable pathological mutations (Table 1). In accordance with our previous report, [6] almost all patients carrying *MEFV* M694I (98.8%) exhibited clinical features of “typical” FMF (Table 1)

**Table 1:** Basic characteristics of the patients with FMF.

	All patients	Clinical form		*MFFV* genotype^†^			
		Typical FMF	Incomplete FMF	M694I -positive	M694I -negative	mutation homo or compound het	mutation hemi or no mutation
Number	n = 258	n = 149	n = 109	n = 85	n = 172	n = 159	n = 99
Age of onset (years, mean±SD)	28.5±18.9	23.6±16.1	35.8±20.6	19.7±12.7	32.9±20.1	26.9±18.2	30.9±20.0
Family history of FMF	53/245 (21.6%)	38/147 (25.9%)	15/98 (15.3%)	25/83 (30.1%)	28/161 (17.4%)	35/152 (23.0%)	18/93 (19.4%)
**Clinical form**							
Typical FMF	149/225 (66.2%)	-	-	**84/85 (98.8%)**	64/172 (37.2%)	104/159 (65.4%)	45/99 (45.5%)
Incomplete FMF	76/225 (33.8%)	-	-	**1/85 (1.2%)**	108/172 (62.8%)	55/159 (34.6%)	54/99 (54.5%)
***MEFV* mutation**							
M694I-positive	85/257 (33.1%)	84/148 (56.8%)	**1/109 (0.9%)**	-	-	69/158 (43.7%)	16/99 (16.2%)
M694I-negative	172/257 (66.9%)	64/148 (43.2%)	**108/109 (99.1%)**	-	-	89/158 (56.3%)	83/99 (83.8%)
mutation homo or compound het	99/258 (38.4%)	45/149 (30.2%)	54/109 (49.5%)	16/85 (18.8%)	83/172 (48.3%)	-	-
mutation hemi or no mutation	159/258 (61.6%)	104/149 (69.8%)	55/109 (50.5%)	69/85 (81.2%)	89/172 (51.7%)	-	-

^†^ Patients with FMF were stratified in two ways according to *MEFV* genotype; (i) presence/absence of a canonical mutation M694I and (ii) homozygosity (homo), compound heterozygosity (compound het) or hemizygosity (hemi) in terms of detectable pathological mutations.

### Effect of *HLA-B* and-*DRB1* on the risk of FMF

Fourteen *HLA-B* alleles (B*07:*02*, B*15:*01*, B*35:*01*, B*39:*01*, B*40:*01*, B*40:*02*, B*40:*06*, B*44:*03*, B*46:*01*, B*48:*01*, B*51:*01*, B*52:*01*, B*54:*01* and B*55:*02*) and 13 *HLA-DRB1* alleles (DRB1*01:*01*, DRB1*15:*01*, DRB1*15:*02*, DRB1*04:*03*, DRB1*04:*05*, DRB1*04:*06*, DRB1*04:*10*, DRB1*12:*01*, DRB1*13:*02*, DRB1*14:*54*, DRB1*08:*02*, DRB1*08:*03* and DRB1*09:*01*) were possessed by 5% or more of the patients or controls. Carriers of B*15:*18* occupied more than 5% of typical FMF. These alleles were examined whether the carrier frequencies were different between patients and controls. Consequently, 4 *HLA-B* (B*39:*01*, B*52:*01*, B*40:*01* and B*44:*03*) and 3 *HLA-DRB1* (DRB1*15:*02*, DRB1*04:*03* and DRB1*08:*02*) alleles were significantly different (Table 2 and S1 Table). The carriers of B*39:*01* were increased in the patients (OR = 3.25, p = 0.0012), whereas those of DRB1*15:*02* were decreased (OR = 0.45, p = 0.00050), satisfying Bonferroni’s correction for multiple statistical test (n = 28, p<0.00179) among them.

**Table 2:** HLA carrier status in the patients with FMF.

*HLA* allele	All patients	Clinical form		*MFFV* genotype			
		Typical FMF	Incomplete FMF	M694I -positive	M694I -negative	mutation homo or compound het	mutation hemi or no mutation
*HLA-B*	n = 257	n = 149	n = 108	n = 85	n = 171	n = 159	n = 98
B*39:*01*	**3.25 (1.53–6.94) p = 0.0012**	2.53 (1.06–6.04) p = 0.030	**4.30 (1.82–10.2) p = 0.00028**	ns	**3.83 (1.73–8.48) p = 0.00037**	3.01 (1.31–6.92) p = 0.0064	3.65 (1.48–9.00) p = 0.0025
B*52:*01*	0.52 (0.34–0.81) p = 0.0030	ns	0.36 (0.19–0.71) p = 0.0020	ns	**0.43 (0.26–0.73) p = 0.0013**	**0.42 (0.25–0.73) p = 0.0013**	ns
B*40:*01*	2.25 (1.24–4.07) p = 0.0060	2.42 (1.25–4.68) p = 0.0069	ns	2.91 (1.38–6.13) p = 0.0034	ns	2.62 (1.38–4.94) p = 0.0023	ns
B*44:*03*	0.63 (0.39–1.00) p = 0.047	0.51 (0.28–0.93) p = 0.025	ns	ns	ns	ns	ns
B*15:*18*	ns	4.69 (1.18–13.9) p = 0.0022	ns	3.68 (1.03–13.1) p = 0.032	ns	3.95 (1.31–11.9) p = 0.0083	ns
B*15:*01*	ns	1.86 (1.03–3.35) p = 0.036	ns	ns	ns	ns	ns
B*35:*01*	ns	ns	1.92 (1.07–3.42) p = 0.025	ns	ns	ns	ns
*HLA-DRB1*	n = 256	n = 148	n = 108	n = 85	n = 172	n = 158	n = 98
DRB1*15:*02*	**0.45 (0.28–0.71) p = 0.00050**	0.55 (0.32–0.93) p = 0.025	**0.32 (0.16–0.66) p = 0.0010**	0.47 (0.23–0.94) p = 0.028	**0.42 (0.24–0.72) p = 0.0013**	**0.36 (0.20–0.64) p = 0.00033**	ns
DRB1*04:*03*	2.97 (1.20–7.32) p = 0.013	3.35 (1.26–8.90) p = 0.010	ns	**4.94 (1.75–13.9) p = 0.00080**	ns	3.43 (1.31–8.97) p = 0.0075	ns
DRB1*08:*02*	2.17 (1.07–4.40) p = 0.027	2.48 (1.14–5.39) p = 0.018	ns	ns	2.44 (1.15–5.19) p = 0.016	**3.19 (1.53–6.66) p = 0.0011**	ns
DRB1*04:*10*	ns	ns	2.75 (1.20–6.28) p = 0.012	ns	ns	ns	2.78 (1.19–6.48) p = 0.013

Odds ratio and its 95% confidence intervals (in parenthesis) for the carriers of *HLA-B* and-*DRB1* alleles were listed when the comparison with the controls gave p<0.05. The results which remained significant after the Bonferroni’s procedure are highlighted in **bold** with their p values: 15 *HLA-B* and 13 *DRB1* alleles, in total 28 *HLA* allele carriers were tested because their frequencies in patents and/or controls were 5% or more; p<0.05/28≈0.00179. ns: not significant, (p ≥ 0.05).

### *HLA* effects on the clinical forms of FMF and *MEFV* genotype

Next, the *HLA* effects were examined for two clinical forms of FMF, typical and incomplete FMF, as well as patients with certain *MEFV* genotypes (Table 2). The precipitating effect of B*39:*01* was observed in incomplete FMF and patients without high-penetrance *MEFV* allele M694I with higher OR and smaller p value than the comparison between FMF and controls (OR = 4.30 and 3.83, p = 0.00028 and 0.00037, respectively), but not in typical FMF or patients carrying M694I. In contrast, the protective effect of DRB1*15:*02* was evident in both typical and incomplete FMF (OR = 0.55 and 0.32, p = 0.025 and 0.0010) and in both carriers and non-carriers of M694I (OR = 0.47 and 0.42, p = 0.028 and 0.0013), while the effect was slightly stronger in incomplete FMF and patients without M694I. The major *HLA* effects were augmented in the patients without canonical *MEFV* variant allele M694I, in accordance with the notion that the lower penetrance in the subset of the disease is owing to the larger contribution of modifiers in the pathogenesis. Additional precipitating alleles such as B*40:*01*, B*15:*18*, B*15:*01*, DRB1*04:*03* and DRB1*08:*02* were significant in typical FMF, whereas B*35:*01* and DRB1*04:*10* were increased only in incomplete FMF.

### Interaction between *HLA* allele*s*

Independence/interaction of HLA factors was evaluated by the comparison of carrier frequencies between subpopulations of patients and controls after stratification by carrier status of another HLA allele of interest. Mantel-Haenszel weighed mean was adopted to estimate an OR for each of 4 *HLA-B* and 3 *HLA-DRB1* significant alleles adjusted for carrier status of the other 6 HLA alleles (Table 3). Among 42 estimations, only OR of B*52:*01* adjusted for DRB1*15:*02* and that of DRB1*15:*02* adjusted for B*52:*01* were profoundly attenuated by adjustment, because B*52:*01* and DRB1*15:*02* were associated non-randomly as one of ancestral HLA haplotypes in the Japanese population.[11] Although it did not reach statistical significance, a point estimation of OR of DRB1*15:*02* remained less than one regardless of the presence or absence of B*52:*01*, but not in *vice versa* (Table 4), suggesting the primary role of DRB1*15:*02* in the protective effect conferred by the B*52:*01*-DRB1*15:*02* haplotype. Adjusted ORs were similar to un-adjusted ORs irrespectively of the carrier status of the second HLA allele for the other combinations, indicating independence of these HLA factors as the modifiers of the disease risk except for B*52:*01*. Further, the unexpected interaction between B*40:*01* and DRB1*15:*02* was also demonstrated by stratification analysis, in which the protective effect of DRB1*15:*02* was completely disabled in the presence of B*40:*01* (Table 4).

**Table 3:** Odds ratio adjusted for carrier status of second *HLA* allele.

*HLA* allele	un-adjusted	adjusted for						
		B*39:*01*	B*52:*01*	B*40:*01*	B*44:*03*	DRB1*15:*02*	DRB1*04:*03*	DRB1*08:*02*
*HLA-B*								
B*39:*01*	**3.25 (1.53–6.94) p = 0.0012**	na	2.88 (1.35–6.18) p = 0.0044	**3.32 (1.58–6.98) p = 0.00081**	**3.25 (1.52–6.97) p = 0.0013**	2.85 (1.33–6.10) p = 0.0048	**3.29 (1.53–7.10) p = 0.0013**	3.18 (1.47–6.88) p = 0.0020
B*52:*01*	0.52 (0.34–0.81) p = 0.0030	0.57 (0.37–0.88) p = 0.010	na	0.54 (0.35–0.83) p = 0.0047	0.51 (0.33–0.80) p = 0.0024	1.00 (0.45–2.24) ns^†^	0.54 (0.35–0.83) p = 0.0043	0.54 (0.35–0.83) p = 0.0042
B*40:*01*	2.25 (1.24–4.07) p = 0.0060	2.31 (1.29–4.15) p = 0.0039	2.13 (1.18–3.82) p = 0.0097	na	2.10 (1.15–3.82) p = 0.013	2.19 (1.23–3.88) p = 0.0062	2.34 (1.29–4.26) p = 0.0041	2.34 (1.28–4.26) p = 0.0043
B*44:*03*	0.63 (0.39–1.00) p = 0.047	0.63 (0.39–1.01) ns	0.61 (0.38–0.97) p = 0.037	0.68 (0.42–1.09) ns	na	0.60 (0.37–0.97) p = 0.035	0.63 (0.39–1.01) ns	0.65 (0.41–1.05) ns
*HLA-DRB1*								
DRB1*15:*02*	**0.45 (0.28–0.71) p = 0.00050**	0.49 (0.31–0.77) p = 0.0019	0.44 (0.19–1.05) ns^†^	**0.45 (0.28–0.71) p = 0.00053**	**0.43 (0.28–0.70) p = 0.00037**	na	**0.47 (0.30–0.74) p = 0.0010**	**0.45 (0.29–0.72) p = 0.00062**
DRB1*04:*03*	2.97 (1.20–7.32) p = 0.013	3.00 (1.19–7.56) p = 0.014	2.83 (1.15–6.97) p = 0.018	3.15 (1.26–7.86) p = 0.0093	2.85 (1.18–6.88) p = 0.015	2.72 (1.08–6.86) p = 0.027	na	2.87 (1.19–6.95) p = 0.014
DRB1*08:*02*	2.17 (1.07–4.40) p = 0.027	2.06 (1.00–4.29) p = 0.047	2.08 (1.03–4.22) p = 0.037	2.28 (1.11–4.68) p = 0.020	2.08 (1.02–4.22) p = 0.039	2.10 (1.04–4.23) p = 0.035	2.12 (1.06–4.26) p = 0.029	na

Odds ratio (and its 95% confidence intervals) for the carriers of *HLA-B* and-*DRB1* alleles were adjusted for carrier status of those alleles. The results which remained significant after the Bonferroni’s procedure are highlighted in **bold** with their p values; p<0.05/28≈0.00179. na: not applicable. ns: not significant, (p ≥ 0.05).

^†^ Because B*52:01 and DRB1*15:02 are in strong linkage disequilibrium in the Japanese population, statistical significance of these tests was severely attenuated.

**Table 4:** Interaction between HLA alleles.

HLA allele / Subpopulation^†^	odds ratio	p	test for homogeneity^‡^
B*52:*01*			
DRB1*15:*02*-negative	0.91 (0.36–2.28)	ns (p = 0.84)	
DRB1*15:*02*-positive	1.36 (0.26–7.22)	ns (p = 0.71)	ns (p = 0.67)
Mantel-Haenszel estimate controlling for DRB1*15:*02*	1.00 (0.45–2.24)	ns (p = 0.99)	
B*40:*01*-negative	0.50 (0.32–0.79)	p = 0.0024	
B*40:*01*-positive	1.47 (0.25–8.57)	ns (p = 0.67)	ns (p = 0.23)
Mantel-Haenszel estimate controlling for B*40:*01*	0.54 (0.35–0.83)	p = 0.0047	
B*40:*01*			
B*52:*01*-negative	1.86 (1.00–3.50)	ns (p = 0.051)	
B*52:*01*-positive	5.44 (0.96–30.8)	p = 0.031	ns (p = 0.24)
Mantel-Haenszel estimate controlling for B*52:*01*	2.12 (1.18–3.82)	p = 0.0097	
DRB1*15:*02*-negative	**1.69 (0.89–3.21)**	**ns (p = 0.10)**	
DRB1*15:*02*-positive	**9.80 (1.73–55.4)**	**p = 0.0015**	**p = 0.043**
Mantel-Haenszel estimate controlling for DRB1*15:*02*	2.19 (1.23–3.88)	p = 0.0062	
DRB1*15:*02*			
B*52:*01*-negative	0.34 (0.07–1.69)	ns (p = 0.17)	
B*52:*01*-positive	0.51 (0.18–1.39)	ns (p = 0.18)	ns (p = 0.67)
Mantel-Haenszel estimate controlling for B*52:*01*	0.44 (0.19–1.05)	ns (p = 0.056)	
B*40:*01*-negative	**0.38 (0.22–0.63)**	**p = 9.7×10** ^**–5**^	
B*40:*01*-positive	**2.20 (0.40–12.2)**	**ns (p = 0.35)**	**p = 0.034**
Mantel-Haenszel estimate controlling for B*40:*01*	0.45 (0.28–0.71)	p = 0.00053	

^†^ The effect of carrier status of *HLA-B* and-*DRB1* alleles were separately calculated in two subpopulations for different carrier status of the second HLA allele. Odds ratio and its 95% confidence interval (in parenthesis) are given. Mantel-Haenszel weighed mean was given (as shown as adjusted odds ratio in Table 3). ns: not significant, (p ≥ 0.05).

^‡^ Test for homogeneity of the effects in two subpopulations. The tests showing differential effects are highlighted in **bold**.

### Response to colchicine treatment

Although the good response to the treatment with oral colchicine administration is one of diagnostic criteria for FMF,[10] a relatively small but not negligible part of the patients are resistant to colchicine-treatment and require alternative anti-inflammatory treatment. Among 170 patients whose clinical data for the response to colchicine were available, 14 patients (8.2%) were clinically classified as non-responders to colchicine-treatment. When the *HLA* alleles significantly associated with FMF, clinical forms of FMF or subgroups of the patients with characteristic *MEFV* genotypes identified above were analyzed (Table 5), B*35:*01* carriers were more likely resistant to colchicine-treatment; 42.9% of 14 treatment-resistant patients and 13.5% of 156 colchicine-responders possessed B*35:*01* allele (OR = 4.82, 95% confidence interval of OR: 1.47–15.8, p = 0.0041), satisfying Bonferroni’s collection for multiple statistical tests (n = 11, p<0.0045).

**Table 5:** HLA carrier status in the poor-responders to colchicine treatment.

*HLA* alleles	Poor responders	Responders	OR	p
*HLA-B*	n = 14	n = 156		
B*39:*01*	4 (28.6%)	17 (10.9%)	3.27 (0.91–11.8)	ns (0.055)
B*52:*01*	1 (7.1%)	26 (16.7%)	0.38 (0.05–3.11)	ns (0.87)
B*40:*01*	1 (7.1%)	23 (14.7%)	0.44 (0.05–3.60)	ns (0.61)
B*44:*03*	2 (14.3%)	21 (13.5%)	1.07 (0.22–5.15)	ns (0.92)
B*15:*18*	0 (0.0%)	7 (4.5%)	0.69 (0.04–12.7)^†^	ns (0.65)
B*15:*01*	0 (0.0%)	23 (14.7%)	0.20 (0.01–3.40)^†^	ns (0.12)
B*35:*01*	**6 (42.9%)**	**19 (13.5%)**	**4.82 (1.47–15.8)**	**0.0041**
*HLA-DRB1*	n = 14	n = 155		
DRB1*15:*02*	0 (0.0%)	19 (12.3%)	0.24 (0.01–4.21)^†^	ns (0.17)
DRB1*04:*03*	0 (0.0%)	11 (7.1%)	0.43 (0.02–7.74)^†^	ns (0.30)
DRB1*08:*02*	4 (26.7%)	17 (10.9%)	0.92 (0.11–7.67)	ns (0.92)
DRB1*04:*10*	2 (14.3%)	6 (3.9%)	4.14 (0.74–23.2)	ns (0.078)

Frequency of which was significantly deviated from that of the controls is highlighted in **bold**.

^†^ OR was obtained by Haldane’s modifications of Woolf’s formula.

## Discussion

### Similarity to other inflammatory diseases with respect to *HLA*-disease association and differential effects of *HLA* classes

As an attempt to identify a modifier gene of FMF, we analyze polymorphisms in the *HLA-B* and-*DRB1* loci because of their possible roles in the determination of antigen specificity in immune response as well as known association with various health conditions including inflammatory diseases, which share certain clinical features with FMF. Consequently, several *HLA-B* and-*DRB1* alleles were demonstrated to be associated with FMF; some of them were reported as risk/protective factor for other diseases. B*39:*01* was associated with severe and sustained osteoarticular manifestations complicated with Brucella infection,[12] and B*27-negative ankylosing spondylitis,[13] suggesting its proinflammatory nature. Indeed, there are accumulating evidences for special ligand binding characteristics with HLA-B*39.[14,15] B*40:*01* which encodes HLA-B60 antigenic specificity was the second most significantly associated *HLA-B* allele with FMF in the present study and has been identified an additional risk factor for ankylosing spondylitis also.[16–18] In contrast, apart from their primary function of antigen presentation to CD4-positive T helper cells in immune response, certain HLA class II alleles play a role in the protection from autoimmunity as in the case of DRB1*13:*02* in systemic lupus erythematosus, rheumatoid arthritis and autoimmune thyroid disease,[19–21] and DRB1*15:*02* in rheumatoid arthritis without anti-citrullinated peptode/protein antibodies (ACPA-negative RA),[22] in Japanese for example, although the underlying biological mechanisms remain to be uncovered. Similarly, the biological implication of DRB1*15:*02* in the protection from FMF is not clear, but non-competitive interaction between B*40:*01* and DRB1*15:*02* (Table 4) was distinctive and can provide a potential clue to the understanding of the contribution of protective HLA in the pathogenesis of FMF.

### Common genetic predisposition to FMF and other inflammatory diseases

It has been reported that the patients with FMF are predisposed to other inflammatory diseases such as ankylosing spondylitis and Behçet disease.[23,24] *MEFV* mutations play a role to increase the risk of these diseases even among the patients without the complication of FMF.[25–28] Although it still remains unclear how the symptoms of periodical fevers of FMF are triggered, the *MEFV* product interacts with cellular components of inflammasome whose activity is augmented by the pathogenic mutations.[29] Therefore, it is reasonable to consider that FMF-associated *MEFV* mutations enhance the cellular responses in the inflammatory diseases which are also associated with certain *HLA* polymorphisms. Uncovering of the common underlying mechanism in the pathogenesis of these inflammatory diseases in the context of *HLA-MEFV* interaction would be helpful to find an effective target of novel therapeutic or preventive measures.

### Predictors of colchicine response

Colchicine is the first choice of the medical treatment of FMF. The response to colchicine is one of remarkable characteristics of FMF and has been a very effective criterion for the differential diagnosis FMF from other inflammatory diseases. In the present study, 14 of 170 patients (8.2%) whose response to colchicine treatment was available were reported as poor responders by care-providing physicians. It has been widely recognized that 5–15% of the patients with FMF do not respond to colchicine.[30] We did not examine *ABCB1* polymorphisms which are associated with colchicine non-responders,[31] in the present study, but six HLA-B*35:*01*-carriers were identified among poor responders (42.9%) giving a significant increase in the carrier frequency in comparison to colchicine responders. The positive predictive value of HLA-B*35:*01*-carriage remained as much as 0.24, but it was a moderately useful predictor of prognosis as assessed by positive likelihood ratio of 4.35.[32] Despite nation-wide collection of FMF in Japan, the evaluation of predictors of colchicine response was made with relatively small number of poor responders only whose clinical documents were available. Therefore the result of the present study should be confirmed by more colchicine poor responders. None the less for this limitation, combination with other prognostic markers such as *ABCB1* SNPs may improve the accuracy of prediction to meet practical demands.

## Conclusions

The differential effects of *HLA* class I and class II alleles on FMF were identified for Japanese population. Modifier genes including HLA explain low penetrance of non-canonical mutations at least in part. Further, HLA-B*35:*01* can be a useful predictive marker for the failure of colchicine treatment.

## Supporting Information

S1 Table Frequency of HLA carriers in the patients with FMF. Frequency of which was significantly deviated from that of the controls is highlighted in bold. ^†^ Patients with FMF were stratified in two ways according to MEFV genotype; (i) presence/absence of a canonical mutation M694I and (ii) homozygosity (homo), compound heterozygosity (compound het) or hemizygosity (hemi) in terms of detectable pathological mutations.
(DOCX)
