# Supplementary material for: Identification of Disease-Promoting HLA Class I and Protective Class II Modifiers in Japanese Patients with Familial Mediterranean Fever
Source: PLoS One. 2015 May 14;10(5):e0125938. doi: 10.1371/journal.pone.0125938 (PMC4431852; doi:10.1371/journal.pone.0125938)
Supplement: S1 Table — Frequency of which was significantly deviated from that of the controls is highlighted in bold. † Patients with FMF were stratified in two ways according to MEFV genotype; (i) presence/absence of a canonical mutation M694I and (ii) homozygosity (homo), compound heterozygosity (compound het) or hemizygosity (hemi) in terms of detectable pathological mutations. (DOCX) [file pone.0125938.s001.docx]

S1_Table. Frequency of HLA carriers in the patients with FMF

| *HLA* alleles | All patients | Clinical form |  | *MFFV* genotype |  |  |  | Controls |
| --- | --- | --- | --- | --- | --- | --- | --- | --- |
|  |  | Typical FMF | Variant FMF | M694I-positive | M694I-negative | mutation homo or compound het | mutation hemi or no mutation |  |
| *HLA-B* | (n=257) | (n=149) | (n=108) | (n=85) | (n=171) | (n=159) | (n=98) | (n=299) |
| *B*39:01* | **10.1%** | **8.1%** | **13.0%** | 7.1% | **11.7%** | **9.4%** | **11.2%** | 3.3% |
| *B*52:01* | **15.2%** | 18.1% | **11.1%** | 20.0% | **12.9%** | **12.6%** | 19.4% | 25.4% |
| *B*40:01* | **13.2%** | **14.1%** | 12.0% | **16.5%** | 11.1% | **15.1%** | 10.2% | 6.4% |
| *B*44:03* | **12.8%** | **10.7%** | 15.7% | 10.6% | 14.0% | 11.9% | 14.3% | 19.1% |
| *B*15:18* | 4.2% | **7.4%** | 0.0% | **5.9%** | 3.5% | **6.3%** | 1.0% | 1.7% |
| *B*15:01* | 14.4% | **16.1%** | 12.0% | 16.5% | 13.5% | 14.5% | 14.3% | 9.4% |
| *B*35:01* | 16.7% | 13.4% | **21.3%** | 16.5% | 17.0% | 17.6% | 15.3% | 12.4% |
| *HLA-DRB1* | (n=256) | (n=148) | (n=108) | (n=85) | (n=172) | (n=158) | (n=98) | (n=299) |
| *DRB1*15:02* | **12.5%** | **14.9%** | **9.3%** | **12.9%** | **11.8%** | **10.1%** | 16.3% | 24.1% |
| *DRB1*04:03* | **6.6%** | **7.4%** | 5.6% | **10.6%** | 4.7% | **7.6%** | 5.1% | 2.3% |
| *DRB1*08:02* | **9.0%** | **10.1%** | 7.4% | 7.1% | **10.0%** | **12.7%** | 3.1% | 4.3% |
| *DRB1*04:10* | 6.6% | 3.4% | **11.1%** | 3.5% | 8.2% | 3.8% | **11.2%** | 4.3% |

Frequency of which was significantly deviated from that of the controls is highlighted in **bold**.

^†^ Patients with FMF were stratified in two ways according to *MEFV* genotype; (i) presence/absence of a canonical mutation M694I and (ii) homozygosity (homo), compound heterozygosity (compound het) or hemizygosity (hemi) in terms of detectable pathological mutations.
